# Fluorescent light energy modulates healing in skin grafted mouse model

**DOI:** 10.1515/med-2021-0329

**Published:** 2021-08-27

**Authors:** Jie Ding, Maiken Mellergaard, Zhensen Zhu, Peter Kwan, Deirdre Edge, Zengshuan Ma, Lise Hebert, Saad Alrobaiea, Takashi Iwasaki, Michael Canova Engelbrecht Nielsen, Edward E. Tredget

**Affiliations:** Wound Healing Research Group, Department of Surgery, Faculty of Medicine and Dentistry, University of Alberta, 161 HMRC, Edmonton, Canada; Department of Veterinary and Animal Sciences, Faculty of Health and Medical Sciences, University of Copenhagen, Denmark; Department of Research and Development, Klox Technologies Europe Ltd, Dublin, Ireland; Department of Research and Development, Klox Technologies Inc., Laval, Canada; Department of Research and Development, Klox R&D Center, Guangdong Klox Biomedical Group Co., Ltd, Room 603, 6/F, Building 8, No. 6, Nanjiang Second Road, Zhujiang Street, Nansha District, Guangzhou, China; Divisions of Plastic and Reconstructive Surgery and Critical Care, 2D2.28 Walter C MacKenzie Health Sciences Centre & Wound Healing Research Group, 161 HMRC, Department of Surgery, University of Alberta, Edmonton, Alberta, Canada

**Keywords:** skin grafting, dermal fibrotic mouse model, fluorescent light energy, photobiomodulation, wound healing

## Abstract

Skin grafting is often the only treatment for skin trauma when large areas of tissue are affected. This surgical intervention damages the deeper dermal layers of the skin with implications for wound healing and a risk of scar development. Photobiomodulation (PBM) therapy modulates biological processes in different tissues, with a positive effect on many cell types and pathways essential for wound healing. This study investigated the effect of fluorescent light energy (FLE) therapy, a novel type of PBM, on healing after skin grafting in a dermal fibrotic mouse model. Split-thickness human skin grafts were transplanted onto full-thickness excisional wounds on nude mice. Treated wounds were monitored, and excised xenografts were examined to assess healing and pathophysiological processes essential for developing chronic wounds or scarring. Results demonstrated that FLE treatment initially accelerated re-epithelialization and rete ridge formation, while later reduced neovascularization, collagen deposition, myofibroblast and mast cell accumulation, and connective tissue growth factor expression. While there was no visible difference in gross morphology, we found that FLE treatment promoted a balanced collagen remodeling. Collectively, these findings suggest that FLE has a conceivable effect at balancing healing after skin grafting, which reduces the risk of infections, chronic wound development, and fibrotic scarring.

## 1 Introduction

Therapy involving skin grafting has significantly advanced over the last decade. Skin grafts are characterized by (1) donor and recipient of tissue and include: autografts (same individual), allografts (same species), and xenografts (different species) and (2) thickness of graft tissue, that is classified as split-thickness human skin grafts (STSGs) or full-thickness skin grafts (FTSGs) consisting of epidermis and part of dermis or epidermis and full thickness dermis, respectively [1]. FTSGs are mostly used for smaller areas and on more exposed body sites, since they need proper vascularity to survive but contract less than STSGs, thus reducing the risk of hypertrophic scar (HTS) development [1].

Skin repair, including rapid wound closure, tissue healing, and reduced scarring after grafting, is essential to generate a fully functional tissue [2]. However, wound healing is a profoundly complex process relying on close collaboration between skin cells, infiltrating immune cells, and the extracellular matrix (ECM) to warrant a balanced healing process [3]. Skin grafting therapies often result in some degree of fibrosis and scarring, thus the development of new effective therapeutic strategies for deep injury to the dermis e.g., in relation to skin grafting are required.

Photobiomodulation (PBM) describes the therapeutic application of light to stimulate regeneration [4,5,6,7]. Indeed, studies have demonstrated that PBM induces anti-inflammatory activity and tissue repair by modulating neovascularization and the early formation of collagen fibers [8,9,10,11,12]. Fluorescent light energy (FLE) is a novel form of PBM consisting of a topical component containing specific chromophores that need activation by a LED lamp, whereby FLE is generated. The chromophores absorb photons from the LED lamp (440–460 nm) and through the phenomenon of Stokes shift, emit lower-energy fluorescent light (500–610 nm) that penetrates intact or wounded tissues [12,13,14]. *In vitro* studies have shown that FLE is superior at inducing collagen production in human dermal fibroblast cell cultures when compared with the blue LED light alone or a lamp mimicking the spectral output of FLE [12]. FLE also significantly downregulates several pro-inflammatory cytokines, including interleukin 6 and tumor necrosis factor α, as well as facilitating early modulation of angiogenesis [12,13,14,15]. Furthermore, FLE has been shown to significantly impact healthy [16], inflamed, and disease-affected skin tissues. The latter being well-documented in clinical trials and case studies, for acne [17,18], rosacea [19,20], acne conglobate and hidradenitis suppurativa [21], acneiforme eruption [22], senile lentigines [23,24], and wound healing [13,25,26,27]. Finally, a series of case studies investigating the effect of FLE treatment on healing of acute second-degree burns showed accelerated wound healing as well as overall improvement of tissue structure in two cases of severe HTS after burn injuries, suggesting that FLE balances wound healing at different stages of the wound healing and remodeling process [28].

Based on these previously reported potent anti-inflammatory and tissue regenerating properties of FLE, we speculated that FLE treatment has the potential to be an effective and preventative therapeutic strategy ensuring rapid and balanced wound healing in patients after skin grafting. Thus, the aim of this study was to investigate the effects of FLE on skin repair after grafting. We used a previously described *in vivo* dermal scar model [29]. This model provides a useful tool for investigating the direct effect of FLE on healing after skin grafting including essential cellular factors associated with dermal fibrotic disorders [30]. Nude mice with human STSGs were treated with either FLE or control LED light and the results were compared with an untreated control (and for some assays normal human skin or human HTS tissue). Wound closure was assessed by monitoring morphological changes and measuring the wound area. Furthermore, histological analysis was used for investigating the healing process focusing on thickness of the dermal layers, vascularity, and re-epithelialization. Finally, collagen deposition, cellular infiltration, and connective tissue growth factor (CTGF) were assessed *in situ*.

## 2 Materials and methods

### 2.1 Animals and study design

The experiment followed Canadian rules for animal treatment and welfare, and the study was approved by Animal Care and Use Committees (ACUCs) of the University of Alberta. A 4-week-old male BALB/c-nu/nu nude mice (*n* = 69) weighing an average of 20 g were purchased from Charles River Laboratories International, Inc. (Wilmington, MA). The mice were first conditioned for 1–2 weeks in the university animal facility and then grafted with split-thickness human grafts from discarded skin flaps of patients undergoing abdominoplasty.

**Figure 1:**
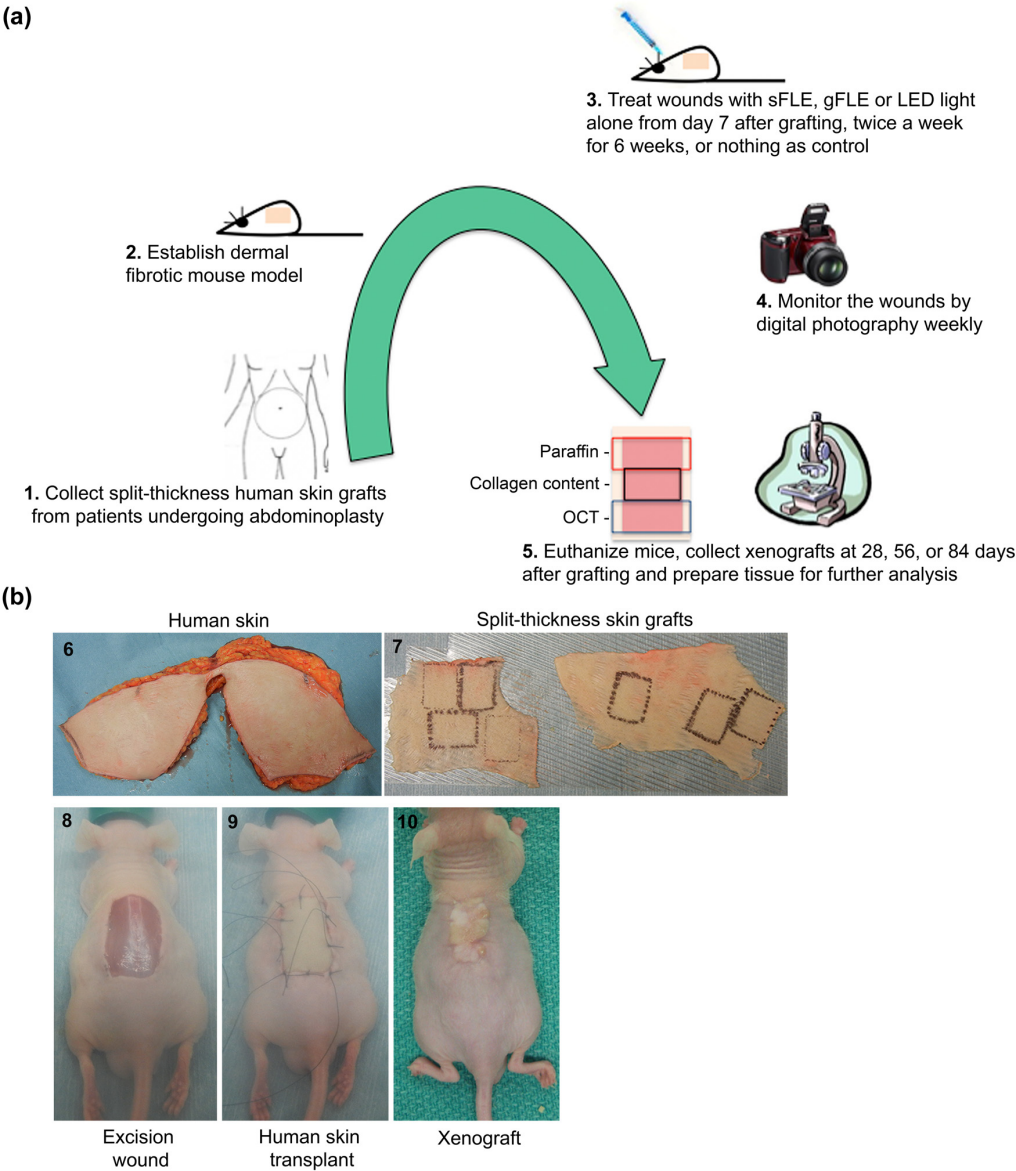
Study procedure and generation of STSG mouse model. (a) Human split-thickness skin grafts (STSGs) were collected from patients who had cosmetic abdominoplasty (1) and transplanted onto full-thickness excisional wounds on the backs of mice (2). The wounds were treated either with sFLE or gFLE plus LED light or LED light alone from Day 7 post-grafting, twice a week for 6 consecutive weeks, or left untreated (3). The wounds were monitored weekly by digital photography (4). The mice were euthanized at day 28, 56, and 84 post-treatment and xenografts were collected for histological analysis. Collected tissue from each mouse was divided into three parts, one was embedded in paraffin (red square), one was prepared and stored for collagen quantification (black square), and one was prepared and stored as OCT blocks (blue square, not used in this study) (5). (b) Representative pictures showing generation of the STSG mouse model used in the study. Human skin from cosmetic abdominoplasty (6) was prepared for STSG grafting on nude mice (7). Excision wound was prepared on recipient mouse (8) prior to STSG grafting (9). Xenograft developed on recipient mouse (10) before initiation of treatment.

Mice were divided into four groups according to the treatments: (1) untreated (Control), (2) LED light alone (Light), (3) solid FLE formulation plus LED light (sFLE), and (4) gel FLE formulation plus LED light (gFLE) (16–18 mice/group and 5–6 mice/group/time point, Table 1). Treatment was performed twice per week from day 7 after grafting and for six consecutive weeks. Wound healing was monitored weekly using digital photography. After 28, 56, or 84 days, the mice were euthanized, using a CO_2_ chamber, and xenografts were collected for histology and biochemistry analysis (Figure 1a).

**Table 1:** Groups and skin donors

Groups	28 days	56 days	84 days	Total
Control	*n* = 5	*n* = 5	*n* = 6	*n* = 16
LED	*n* = 6	*n* = 6	*n* = 6	*n* = 18
sFLE	*n* = 6	*n* = 6	*n* = 6	*n* = 18
gFLE	*n* = 5	*n* = 6	*n* = 6	*n* = 17
Total	*n* = 22	*n* = 23	*n* = 24	*n* = 69
Skin donor	*n* = 1	*n* = 2	*n* = 2	*n* = 5

### 2.2 Preparation of human STSGs

Abdominal human skin tissues were collected from five white female patients (19, 42, 50, 55, and 56 years old) who had cosmetic abdominoplasty. STSGs were harvested *ex vivo* using a Padgett electric dermatome (Padgett Instruments, INC., Kansas City, MO) set at 0.03 cm. They were cut into pieces of 2.0 cm × 1.5 cm (Figure 1b) and kept in sterile normal saline for grafting. The Health Research Ethics Board of the University of Alberta Hospital approved the patient protocol. All human subjects who provided tissues gave written informed consent, which was documented in the patient’s health record before participation in the study.

### 2.3 Establishment of human dermal fibrotic mouse model

A full-thickness excisional wound (2.0 cm × 1.5 cm) was made on the back of each mouse under isoflurane anesthesia. A human STSG, randomized to the four groups, was transplanted onto the wound and secured with sutures (Figure 1b). The surgical site was then covered with a non-adherent petrolatum (Xeroform™, Covidien, Mansfield, MA) and gauze in a tie-over bolster dressing for 7 days after grafting to ensure adherence of the human skin graft to the wound bed of the mouse tissue, as previously described [31].

### 2.4 FLE treatment

Treatment was initiated on day 7 after grafting when the dressing was removed. Mice were treated under anesthesia via nasal halothane and treated twice per week for 6 consecutive weeks. Two different FLE systems (Klox Technologies Inc., Québec, Canada) were used. The gFLE was prepared by mixing a chromophore gel with a hydro-carrier gel immediately before use and the mixed gFLE was applied as a thick layer of 2 mm on the wound. The sFLE (solid membrane form of the FLE gel containing chromophores) was cut into pieces of 1.5 cm^2^ × 2 cm^2^ and placed on the wound. Both the FLE systems emit similar FLE emission spectra between 500 and 610 nm, as previously described [13,15]. Illumination was performed for 5 min at a distance of 5 cm from the wound. The LED lamp (Klox Technologies Inc., Laval, Canada) used to illuminate the chromophores in the gel or membrane delivers a non-coherent blue light with a single peak wavelength and a maximum emission between 440 and 460 nm. The power density of the Klox lamp (Klox Technologies Inc., Laval, Canada) was measured using a multifunctional spectroradiometer system (SP-100, Orb Optronix inc., Kirkland, WA, USA) with a spectral range between 380 and 780 nm. The wavelength binning resolution of the optical system was 1 nm. The optical densities of the lamp were determined at a 5 cm distance. The power output of the Klox lamp is certified to be within 120–125 mW/m^3^ and the fluency between 33 and 45 J/cm^2^ at 5 min treatment [12,13,14,15].

### 2.5 Digital photography

Wounds were monitored morphologically by digital photography every week after grafting to document wound healing and scar formation. A scale was embedded in each photo to allow for direct measurements.

### 2.6 Quantification of wound closure

Wound closure was assessed and quantified at day 28, 56, or 84 post-treatment by measuring the wound area and normalizing measurements according to a scale embedded in each picture using ImageJ (National Institutes of Health, Bethesda, MD). Wound closure was presented as percentage of wound area.

### 2.7 Evaluation of re-epithelialization in xenografts

Xenografts were collected after the mice were euthanized using a CO_2_ chamber at day 28, 56, or 84 post-treatment and prepared for histological analysis. Sections were stained with hematoxylin and eosin (H&E), imaged using a light microscope (Nikon Canada Inc., Mississauga, ON, Canada), and analyzed according to a scale in each image. Epithelialization (integrity of epidermal layer) and re-epithelialization (upward migration of epithelial cells) were assessed in the H&E images and examined against sections from a skin sample (non-grafted) from one donor at each time point to compare grafted tissue with normal human skin tissue (NS samples).

### 2.8 Quantification of epidermal and dermal thicknesses and vascularity in xenografts

ImageJ was used to measure epidermal and dermal thicknesses using high-power magnification in H&E images. Epidermal thickness was defined as the distance from skin surface down to the bottom of epidermal papillae and dermal thickness as the distance between the epidermal–dermal junction and the dermal–adipose layer junction, as previously described for dermal thickness [32]. Three measurements were taken per sample, two adjacent to the wound site at 50 μm on each side and one in the middle of each section. The vascularity was assessed by counting the number of blood vessels in 20× magnification views taken diagonally from top left to bottom right in the dermis, in five random high-power fields (HPFs) for each animal.

### 2.9 Quantification of collagen in xenografts

Collagen content was quantified in the xenografts by 4-hydroxyproline analysis using liquid chromatography/mass spectrometry (LC/MS), as previously modified and described [33]. 5–10 mg of xenograft tissues (wet weight) were freeze-dried overnight. The dried tissues were then hydrolyzed in 6 N hydrochloric acid (HCL) solution at 110°C, resulting in collagen being cleaved into its component amino acids and 4-hydroxyproline being released from the collagen protein. Then, a known amount of *N*-methyl-proline was added as an internal standard along with the *n*-butyl-ester reagent for derivatization. After the mixture was dried under vacuum, the determination of 4-hydroxyproline was performed using an HP Hewlett Packard 1100 LC linked to 6130 MS (Agilent Technologies, Santa Clara, CA) selective detector monitoring the ions 188 (*N*-butyl-ester of 4-hydroxyproline) and 186 (*N*-butyl-ester of *N*-methyl-proline). Each sample was run in triplicate, and data are displayed as ng of 4-hydroxyproline per mg dry tissue obtained by reference to a standard curve.

### 2.10 Collagen assessment

Paraffin-embedded sections were deparaffinized and hydrated after being washed twice in acidified water, dehydrated and mounted before staining in picrosirius red (Abcam, Toronto, ON, Canada) for 1 h. Picrosirius red stain provides a simple, specific, and sensitive method to localize collagen in the tissues by reacting with sulfonic acid groups present in the collagen molecule [34]. Collagen bundles appear as green, red, or yellow on a black background allowing quantitative morphometric analysis under polarized light. Collagen orientation was evaluated by Fourier analysis using ImageJ software (ImageJ v.1.51 u NIH, USA), represented by the collagen orientation index (COI). Briefly, the original single collagen bundle in each picrosirius red image taken under polarized microscope was converted to a representation in the frequency domain by ImageJ software. The threshold was adjusted to make it clear. The width and length of the representation was measured and the COI was calculated using the following equation: 1 − (short axis/long axis), as previously described [35], and presented as arbitrary units (a.u.). The collagen networks in xenografts were compared with NS and HTS from patients recovered from burn injury. Random collagen bundle tissue like NS has an index of 0 whereas the parallel organization seen in tissue like HTS leads to an index closer to 1.

### 2.11 Myofibroblast and CTGF assessment

To detect involvement of myofibroblasts and fibrotic factors in scar formation, xenografts were stained for alpha smooth muscle actin (αSMA) and CTGF after paraffin-embedded sections were deparaffinized and hydrated. Antigen retrieval was performed on paraffin sections using 0.05% of trypsin in PBS for 15 min. The sections were blocked with serum for 5 min at room temperature (RT), rinsed, and incubated with primary antibodies of monoclonal rabbit anti-αSMA (EMD Millipore, Billerica, MA) and polyclonal goat anti-CTGF (Santa Cruz Biotechnology, Dallas, TX) for 1 h at RT. Secondary antibodies used were biotinylated anti-rabbit IgG (Vector Laboratories, Burlingame, CA) for αSMA staining or biotinylated anti-goat IgG (Dako, Glostrup, Denmark) for CTGF staining for 1 h at RT. Detection was done using VECTASTAIN Elite ABC Reagent and Vector DAB substrate (Vector Laboratories, Burlingame, CA) and counterstained in hematoxylin. Finally, the sections were mounted after dehydration and imaged using an optical microscope.

Myofibroblasts and endothelial cells around blood vessels were all stained by anti-αSMA antibody and were distinguished based on location and morphology. Myofibroblasts were counted in five HPFs/animal. CTGF was qualified in the sections by brown staining using ImageJ software. Color devolution was done by choosing H&E DAB plugin on a CTGF stained image, subtracting background by adjusting threshold, and measuring the CTGF stained area, this was expressed as a percentage of the image area.

### 2.12 Mast cell assessment

Xenograft sections were stained in toluidine blue (Fisher Chemical, Geel, Belgium) working solution for 2–3 min after deparaffinization and hydration. After they were washed with distilled water, the sections were then dehydrated quickly through 95 and 100% of ethyl alcohol (Commercial Alcohols, Brampton, ON, Canada) and cleared in xylene. Finally, the stained sections were mounted for observation by light microscopy. Toluidine blue is a cellular dye with high affinity for acidic tissue components such as heparin- and histamine-rich metachromatic granules in the cytoplasm of mast cells, staining mast cells red-purple and the background blue [30]. The red-purple mast cells were counted in five HPFs/animal.

### 2.13 Statistical analysis

Data are presented as mean value ± SE. Group data (5–6 mice/group, Table 1) at each time point was analyzed using GraphPad Prism version 9.0.0 (GraphPad Software), and each treatment group was statistically compared with the control group using two-way ANOVA with Dunnett´s multiple comparison test, with the exception of the NS versus HTS comparison in Figure 5c, which was done using an unpaired, two-tailed *t*-test. A *p*-value ≤ 0.05 was considered statistically significant.

## 3 Results

### 3.1 FLE treatment modulated wound closure after human STSG transplantation on mice

**Figure 2:**
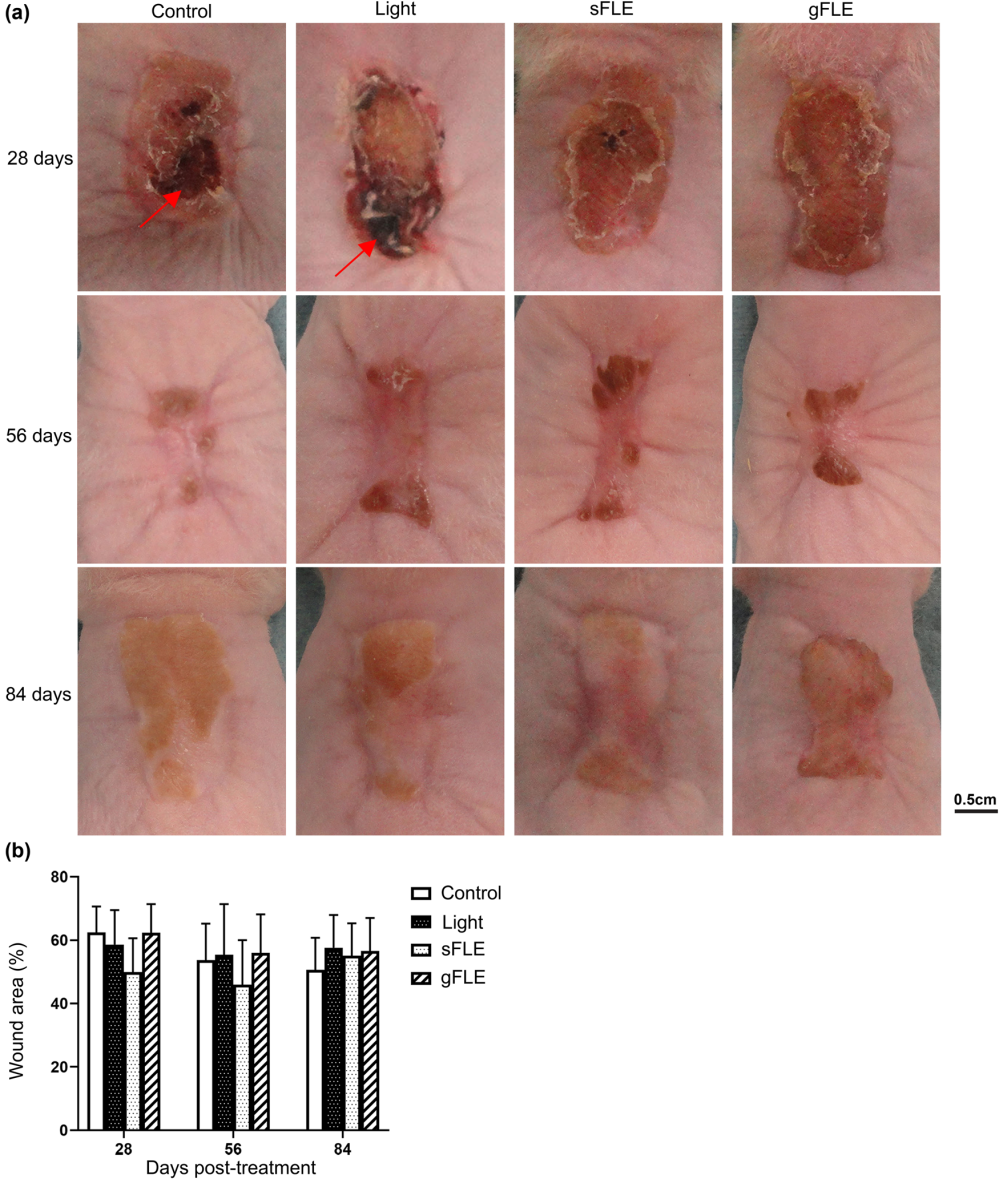
sFLE accelerated wound closure after skin grafting. The wounds were treated with LED light alone (Light) or two different FLE treatments plus LED light (sFLE or gFLE) twice a week for six consecutive weeks, or left untreated (Control). (a) Representative pictures showing wounds monitored by digital photography every week after grafting. Pictures show appearance of healing at day 28 (upper row), 56 (middle row), and 84 (lower row) after last treatment. Scale bar, 0.5 cm. (b) Wound closure was quantified as percentage of measured wound area and presented as bar graphs showing mean value ± SE.

Wound closure was monitored after human STSGs were transplanted onto full-thickness excisional wounds on the back of the mice. Standard morphology assessment showed that FLE treatment (sFLE and gFLE) accelerated wound closure 28 days after treatment when compared with control or light treatment alone (Figure 2a, upper row). Quantification of wound closure by measuring the wound area revealed a reduction in the wound size of the sFLE treated group (sFLE: 49.9 ± 10.7%) compared with the control group (Control: 62.4 ± 8.2%) at 28 days post-treatment (Figure 2b), although this reduction was not significant (Figure S1). Furthermore, complete healing of the graft edges were observed, scabs were almost completely gone, and smooth epithelium covered the entire wound on FLE-treated mice (Figure 2a, upper row). Meanwhile, in both control groups (Control and Light), large scabs remained at 28 days after treatment (indicated by red arrows in Figure 2a, upper row), suggesting incomplete healing of the wounds and delayed wound healing compared with FLE-treated mice at this time point. Over time, all wounds healed and started to contract (Figure 2a, middle row). Moreover, parts of the developing scars became thicker, shiny, and raised in all of the groups 56 days after treatments (Figure 2a, middle row). At day 84 after treatment, the grafts had expanded and developed HTS-like tissues, clearly distinguishable from the surrounding mouse skin. Although FLE accelerated wound healing at 28 days after treatment, there was no obvious superficial difference in scar morphology at day 56 or 84 after treatment (Figure 2a), or in the measured wound area at day 56 or 84 (Figure 2b and Figure S1).

### 3.2 FLE treatment induced re-epithelization and reduced epidermal thickness after skin grafting

**Figure 3:**
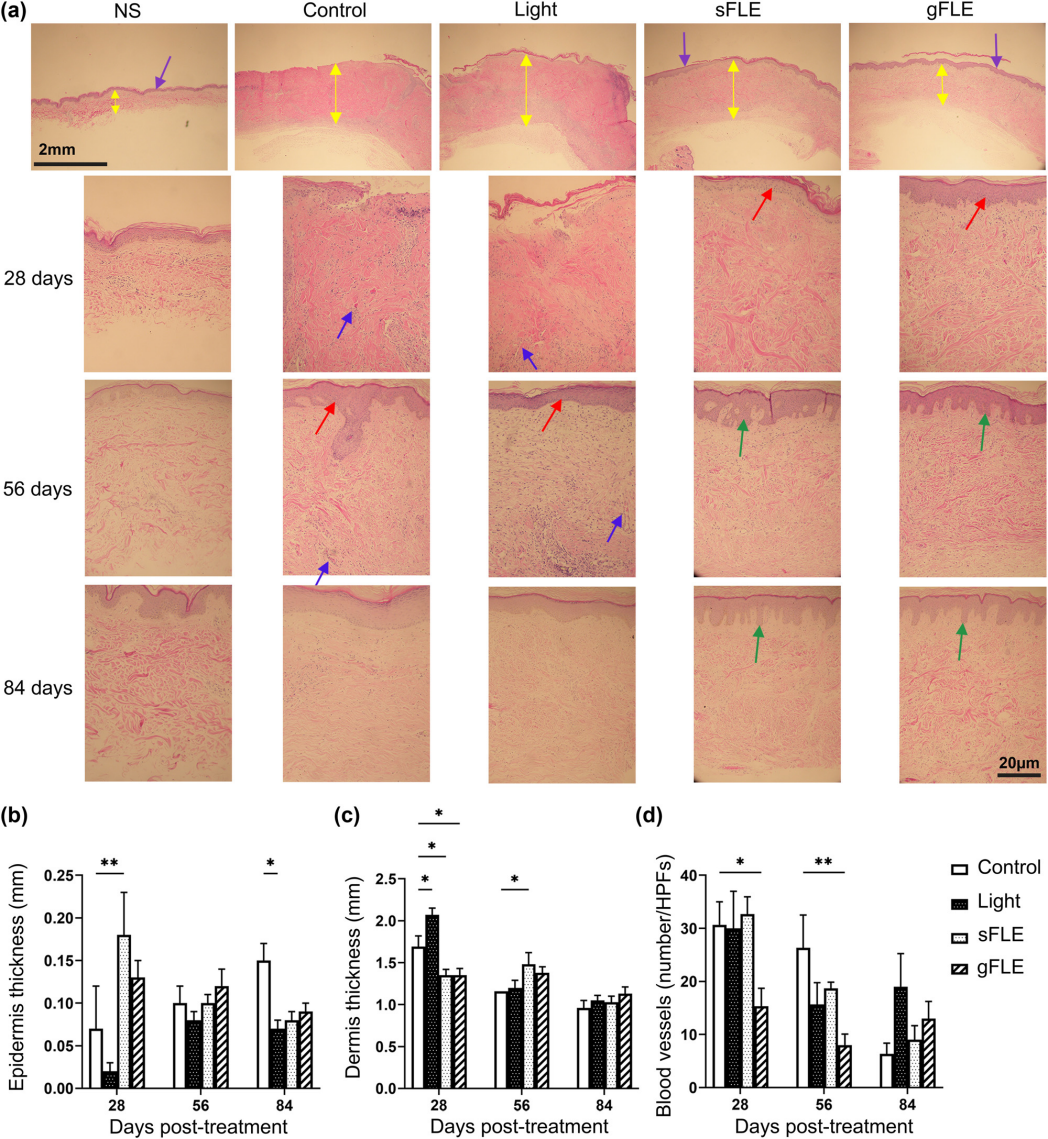
FLE promoted reepithelization, and reduced epidermal thickness and vascularity. (a) Representative images showing H&E staining of human normal skin (NS) and xenografts harvested from mice treated with LED light alone (Light) or two different FLE treatments plus LED light (sFLE or gFLE) at day 28, 56, and 84 post-treatment, or untreated (Control). Assessment of epidermis thickness (purple arrows), dermis thickness (yellow arrows), blood vessels (blue arrows), epithelialization (red arrows), and rete ridges (green arrows) were observed, measured, and counted. Scale bar, 20 µm. (b–d) Quantified data are presented as bar graphs showing the mean value ± SE of (b) epidermis thickness, (c) dermis thickness, and (d) blood vessel numbers/HPFs. *p ≤ 0.05; **p ≤ 0.01.

Re-epithelialization is an essential measure of wound healing that is often impaired in chronic wounds, and is therefore used as a defining parameter for successful wound closure [36]. Since FLE treatment seemingly accelerated early wound closure, we next examined re-epithelialization. Light microscopy of xenograft sections stained with H&E revealed that after 28 days, FLE treatment (sFLE and gFLE) induced a flat epidermal layer with complete re-epithelialization (Figure 3a, red arrows) compared with NS or control groups (Control or Light) where re-epithelialization was delayed and only clearly appearing after 56 days (Figure 3a, red arrows). Interestingly, over time the epithelium expanded and rete ridges were formed in the groups treated with FLE (Figure 3a, green arrows), but not in the two control groups (Figure 3a). Next the thickness of the epidermis (Figure 3a, purple arrows) and the dermis (Figure 3a, yellow arrows) were measured and quantified. All three PBM-treated groups showed reduced epidermal thickness of xenografts compared with the control group at 84 days post-treatment, although this reduction was only statistically significant in the light-treated group (Control: 0.15 ± 0.02 mm vs Light: 0.07 ± 0.01 mm; *p* = 0.0463) (Figure 3b and Figure S2a), while PBM treatments already significantly diminished the dermal thickness at 28 days post-treatment (Light: 2.07 ± 0.08 mm; *p* = 0.0106, sFLE: 1.35 ± 0.07 mm; *p* = 0.0246, and gFLE: 1.35 ± 0.08 mm; *p* = 0.0328) compared with the control group (Control: 1.69 ± 0.13 mm) (Figure 3c and Figure S2b). Besides, sFLE significantly increased dermal thickness at 56 days (sFLE: 1.48 ± 0.14 mm vs Control: 1.16 ± 0.00 mm; *p* = 0.0363), and no significant difference in dermal thickness between groups was observed at day 56 or 84 post-treatment (Figure 3c and Figure S2b).

As angiogenesis is vital for wound healing [37,38], we evaluated the number of blood vessels in the collected xenografts (Figure 3a, blue arrows). Given that STSGs does not contain a dermis when transplanted, all blood vessels in the xenograft are newly formed. Compared with the control group, we found a significant reduction in blood vessel numbers after treatment with gFLE during the first 28 (gFLE: 15.33 ± 3.38 number/HPFs vs Control: 30.67 ± 4.33 number/HPFs; *p* = 0.0466) and 56 days (gFLE: 8.00 ± 2.08 number/HPFs vs Control: 26.33 ± 6.17 number/HPFs; *p* = 0.0097) after treatment (Figure 3d and Figure S2c).

Collectively, these data suggest that FLE treatment enhances wound closure and re-epithelialization while reducing scar thickness and neovascularization within 28 days after injury.

### 3.3 FLE treatment modulated collagen deposition and remodeling after skin grafting

**Figure 4:**
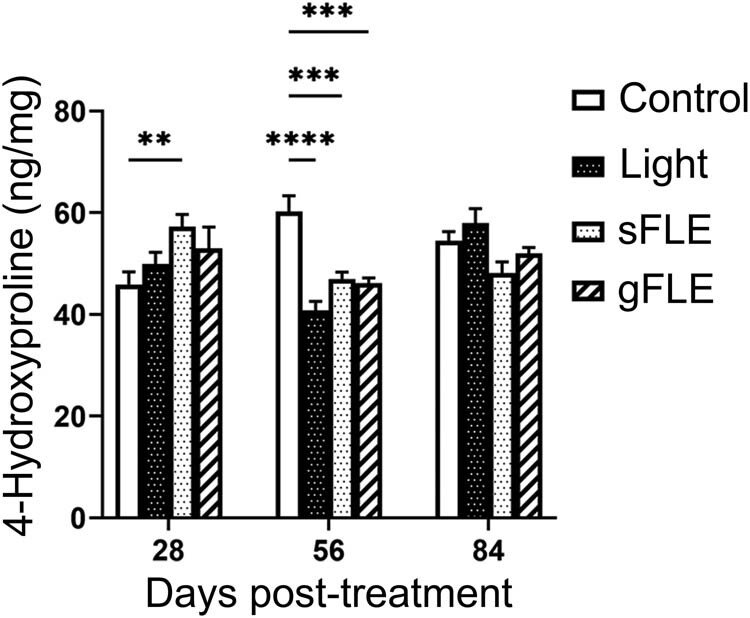
FLE modulated collagen production. Collagen production in xenografts harvested from mice treated with LED light alone (Light) or two different FLE treatments plus LED light (sFLE or gFLE) at day 28, 56, and 84 post-treatment, or untreated (Control) was quantified by 4-hydroxyproline assessment. Bar graphs present the mean value ± SE of 4-hydroxyproline production ng/mg of dry tissue referring to a standard curve. Experiments were performed in triplicate for each sample. **p ≤ 0.01; ***p ≤ 0.001; ****p ≤ 0.0001.

Collagen is a key component of the ECM and a balanced decomposition is vital for ensuring tissue healing without causing fibrosis [35,39]. Quantification of collagen in the xenografts demonstrates that collagen deposition was significantly induced by sFLE (sFLE: 57.21 ± 2.46 ng/mg; *p* = 0.0036) treatment at day 28 compared with the control group (Control: 45.88 ± 2.46 ng/mg), and reduced in all three groups of mice treated with PBM (Light: 40.76 ± 1.80 ng/mg; *p* < 0.0001, sFLE: 46.98 ± 1.31 ng/mg; *p* = 0.0007, and gFLE: 46.15 ± 1.00 ng/mg; *p* = 0.0003) compared with the control group (Control: 60.18 ± 3.15 ng/mg) at day 56 post-treatment, while no difference was observed at day 84 (Figure 4 and Figure S3).

**Figure 5:**
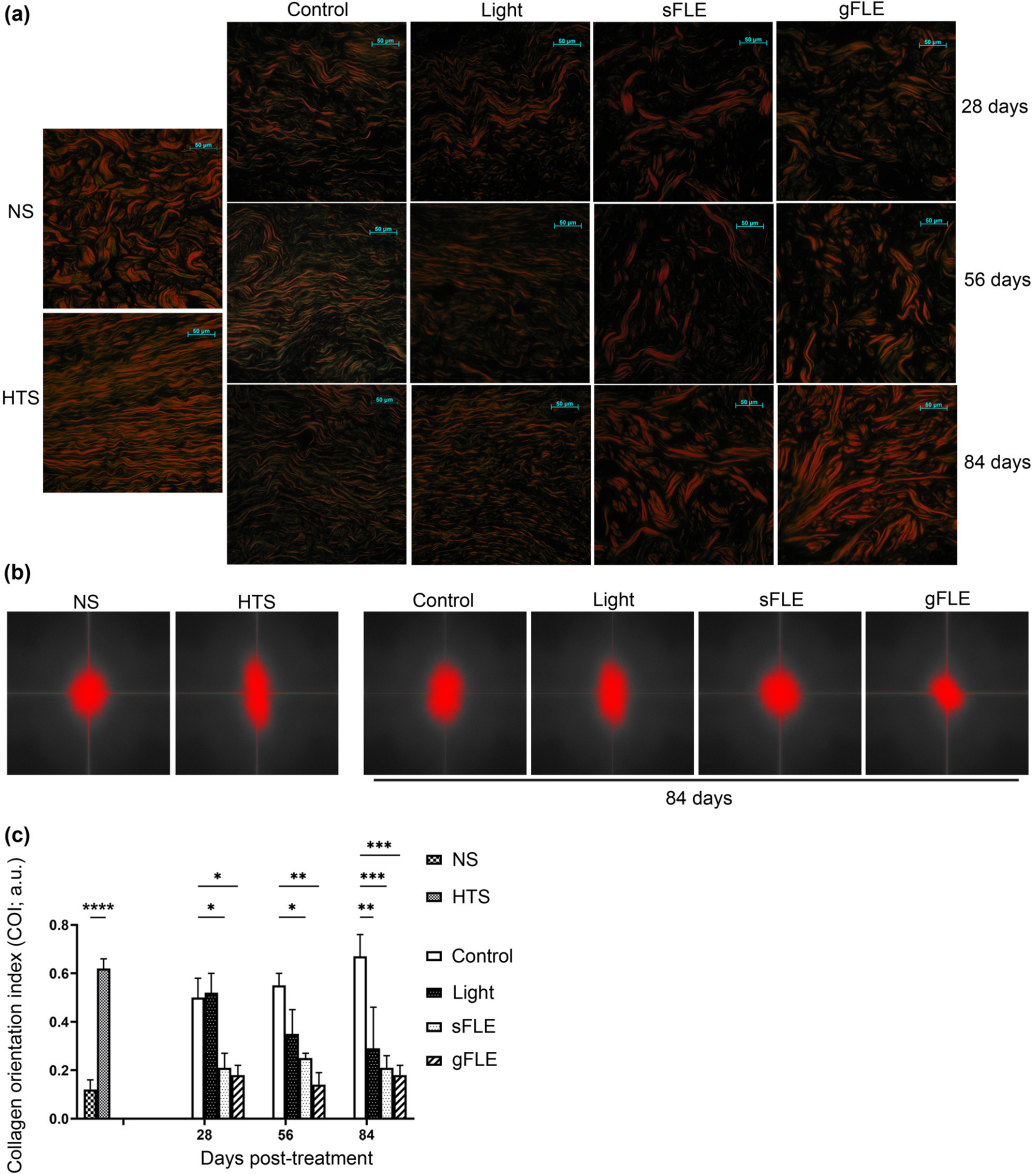
FLE improved collagen orientation and remodeling. (a) Representative images showing picrosirius red staining of human hypertrophic scar (HTS), site-matched human normal skin (NS), and xenografts harvested from mice treated with LED light alone (Light) or two different FLE treatments plus LED light (sFLE or gFLE) at day 28, 56, and 84 post-treatment, or untreated (Control) was performed to appraise collagen networks. Under polarized light, collagen bundles appeared green, red, or yellow. Scale bar, 50 µm. (b) Representative images of collagen orientation evaluated by Fournier analysis in the xenografts harvested from mice at day 84 post-treatment. (c) Bar graphs present the mean value ± SE of the COI calculated using the equation: 1 − (short axis/long axis) as arbitrary units (a.u.). *p ≤ 05; **p ≤ 0.01; ***p ≤ 0.001; ****p ≤ 0.0001.

Next the structure of the collagen network was assessed using picrosirius red staining. The orientation of collagen molecules is an important determinant of their functionality in connective tissues [40]. It is furthermore known that in scar tissue, the collagen network differs from the collagen structure in normal skin, in which collagen forms a “basket-weave” structure with perpendicular collagen fibers [39]. First, we saw that the structure of collagen bundles in human HTS tissue consisted of thin collagen fibers and smaller bundles that aligned parallel with the epidermis compared with human NS tissue (Figure 5a, left column). In xenografts of control mice, the collagen bundle structure resembled that of human HTS (Figure 5a, Control and left column). Conversely, in xenografts from mice treated with FLE, collagen structure resembled the arrangement seen in human NS (Figure 5a, sFLE, gFLE, and left column).

Finally, collagen organization was quantified using the COI by Fourier analysis [41,42]. The short and long axes of the collagen bundles were measured for each scar (Figure 5b and Figure S4a) and the COIs were calculated (Figure 5c and Figure S4b). First, the COI of NS (NS: 0.12 ± 0.04 a.u.) was significantly lower compared with HTS (HTS: 0.62 ± 0.04 a.u.; *p* < 0.0001). Furthermore, data demonstrated a significant decrease in COI in FLE-treated mice (sFLE: 0.21 ± 0.06 a.u.; *p* = 0.0423, and gFLE: 0.18 ± 0.04 a.u.; *p* = 0.0300) compared to that in control group (Control: 0.50 ± 0.08 a.u.) at day 28, day 56 (Control: 0.55 ± 0.05 a.u. vs sFLE: 0.25 ± 0.02 a.u.; *p* = 0.0341, or gFLE: 0.14 ± 0.05 a.u.; *p* = 0.0025), and for all PBM-treated groups on day 84 (Control: 0.67 ± 0.09 a.u. vs Light: 0.29 ± 0.17 a.u.; *p* = 0.0034, sFLE: 0.21 ± 0.05 a.u.; *p* = 0.0004, or gFLE: 0.18 ± 0.04 a.u.; *p* = 0.0001) (Figure 5c and Figure S4b). Furthermore, COI of xenografts from FLE-treated mice resembled COI of NS tissue (Figure 5c), suggesting an improvement in collagen orientation and remodeling mediated by FLE treatments.

### 3.4 FLE treatment reduced levels of myofibroblasts, mast cells, and CTGF in xenografts

**Figure 6:**
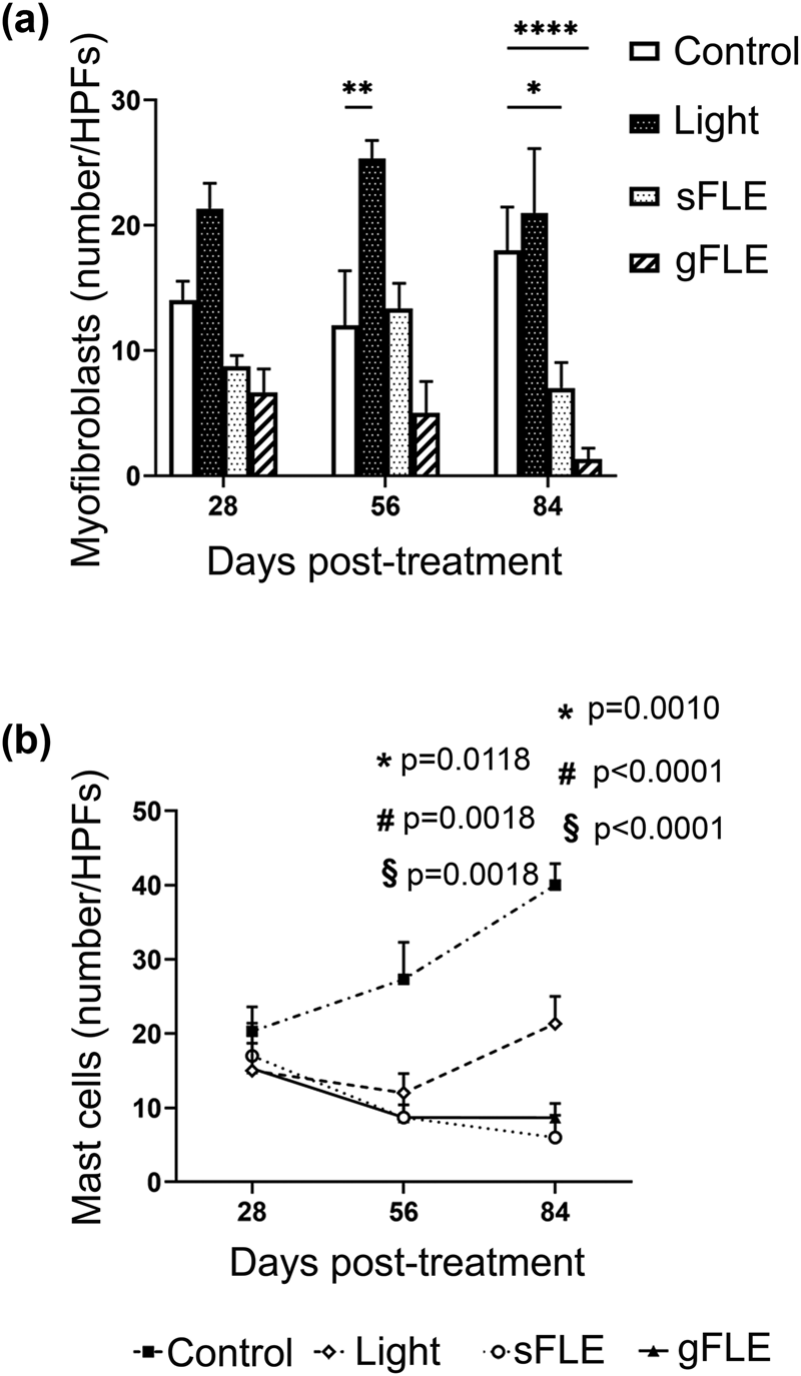
FLE reduced myofibroblast formation and mast cell accumulation. (a) Bar graphs present the mean value ± SE of myofibroblasts in 5 HPFs in the xenografts harvested from mice treated with LED light alone (Light) or two different FLE treatments plus LED light (sFLE or gFLE) at day 28, 56, and 84 post-treatment, or untreated (Control). *p ≤ 0.05; **p ≤ 0.01; ****p ≤ 0.0001. (b) Graph presents the mean value ± SE of mast cells in 5 HPFs in the xenografts harvested from mice treated with LED light alone (Light) or two different FLE treatments plus LED light (sFLE or gFLE) at day 28, 56, and 84 post-treatment, or untreated (Control).

Myofibroblasts play a crucial role in wound healing via ECM synthesis and wound contraction. However, excessive proliferation of differentiated αSMA-expressing myofibroblasts is associated with increased fibrosis [43]. Hence, the impact of FLE on myofibroblasts was investigated by quantifying these cells in the dermis of xenografts by determining αSMA staining (exemplified in Figure S5a). Our results demonstrate that FLE treatment decreased the number of myofibroblasts in xenograft tissues (Figure 6a). Although these findings were not significant on day 28 or 56 (Figure 6a and Figure S5b), day 84 findings showed that both sFLE and gFLE significantly reduced myofibroblast numbers in the tissue post-treatment (Control: 18.0 ± 3.46 cells/HPFs vs sFLE: 7.0 ± 2.04 cells/HPFs; *p* = 0.0114, or gFLE: 1.33 ± 0.88 cells/HPFs; *p* < 0.0001), while treatment with LED light alone significantly promoted myofibroblasts within the first 56 days (Control: 12.0 ± 4.36 cells/HPFs vs Light: 25.3 ± 1.45 cells/HPFs; *p* = 0.0029) after treatment when compared with the control group (Figure 6a and Figure S5b).

Recent studies have highlighted the importance of mast cells in the release of various mediators that affect cell proliferation and collagen remodeling during wound healing, with high numbers of activated mast cells associating with scarring [44]. The number of mast cells were therefore analyzed in xenografts after FLE treatment. Toluidine blue staining was done to assess mast cell numbers in grafted tissue (exemplified in Figure S6a). PBM treatment significantly reduced mast cell numbers in the xenografts at day 56 (Control: 27.3 ± 5.00 cells/HPFs vs Light: 12.0 ± 2.60 cells/HPFs; *p* = 0.0118, sFLE: 8.70 ± 1.70 cells/HPFs; *p* = 0.0018, or gFLE: 8.70 ± 3.50 cells/HPFs; *p* = 0.0018) and day 84 (Control: 40.0 ± 2.90 cells/HPFs vs Light: 21.3 ± 3.70 cells/HPFs; *p* = 0.0010, sFLE: 6.00 ± 3.00 cells/HPFs; *p* < 0.0001, or gFLE: 8.67 ± 1.90 cells/HPFs; *p* < 0.0001) post-treatment compared with the control group (Figure 6b and Figure S6b). Interestingly, the light-mediated down-modulation observed at day 56 had partly increased at 84 days after treatment (Figure 6b and Figure S6b).

**Figure 7:**
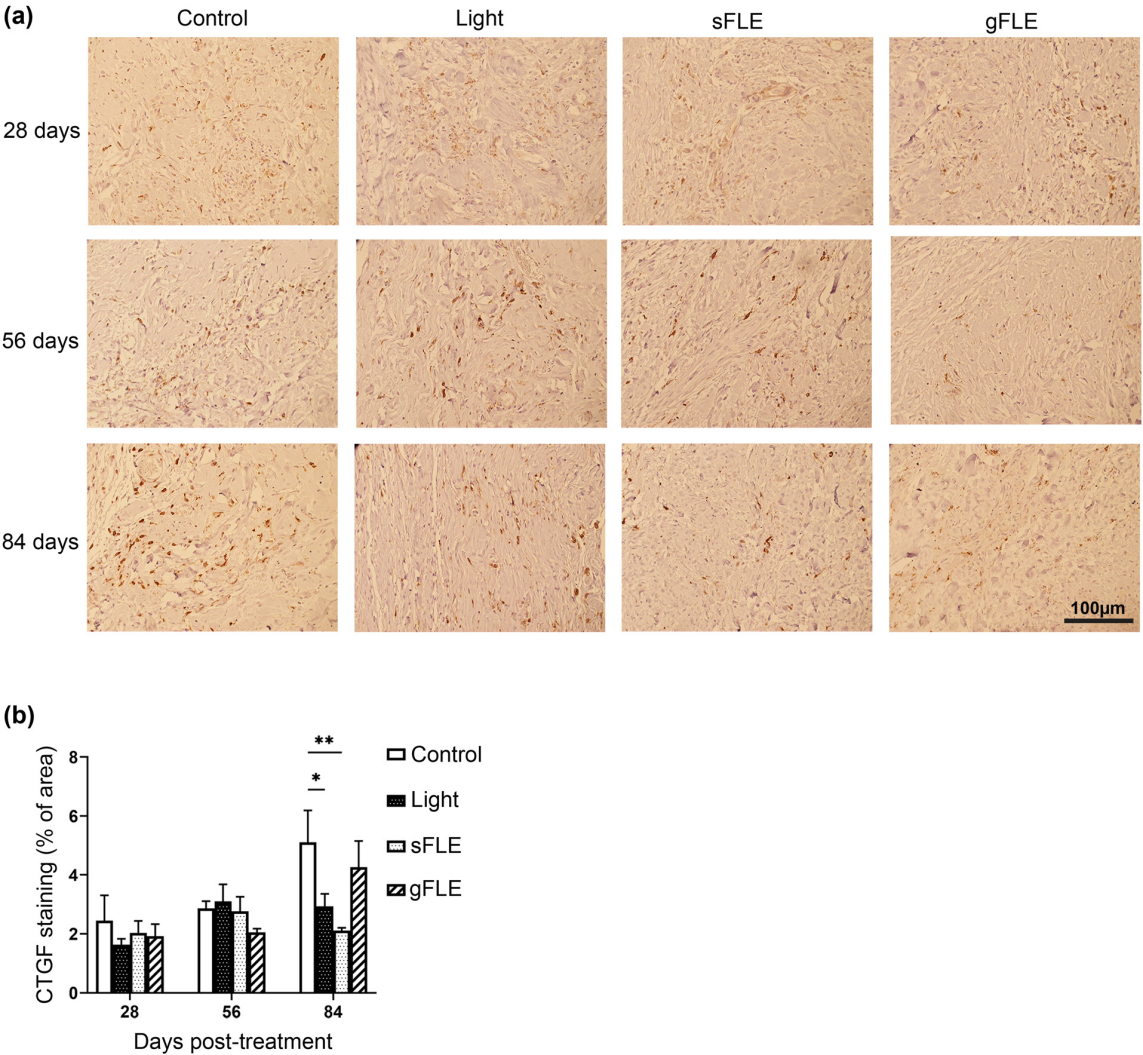
FLE decreased fibrotic factor production. (a) Representative images showing connective tissue growth factor (CTGF) staining (dark brown) in xenografts harvested from mice treated with LED light alone (Light) or two different FLE treatments plus LED light (sFLE or gFLE) at day 28, 56, and 84 post-treatment, or untreated (Control). Scale bar, 100 µm. (b) Bar graphs present the mean value ± SE of CTGF stained area (% of area). *p ≤ 0.05; **p ≤ 0.01.

Finally, expression of CTGF, which is an important growth factor known to be overexpressed and involved in fibrosis and scar formation [45], was assessed in the graft tissue (Figure 7a). CTGF expression was not significantly modulated in any of the groups at 28 and 56 days post-treatment (Figure 7b and Figure S7). However, CTGF expression was significantly reduced by Light and sFLE (Light: 2.93 ± 0.43% of area; *p* = 0.0220 or sFLE: 2.11 ± 0.10% of area; *p* = 0.0011) compared with control group (Control: 5.11 ± 1.08% of area) at day 84 (Figure 7b and Figure S7).

Together these data suggest that FLE-treatment modulates wound closure by accelerating re-epithelialization, while normalizing the collagen organization, blood vessel formation, and reducing the risk of scarring through reduced numbers of myofibroblasts, mast cells, and CTGF levels.

## 4 Discussion

Ensuring swift and balanced wound healing following skin grafting is essential to reduce the risk of infection which can lead to prolonged inflammation, increasing the risk of chronic wounds, fibrosis, and severe scarring [1]. This study sought to investigate the effect of FLE therapy in wound healing after grafting, based on the hypothesis that FLE represents a novel approach to ensure balanced healing with reduced risk of scarring for graft patients.

We used our previously described modified mouse scar model [29] that offers the advantages of low cost, easy manipulation, and short time frame for scar formation and remodeling, compared with several other dermal wound models, including the *in vivo* human scratch wound model [46], rabbit ear [47], or red Duroc pig ding [48]. Our model [29], in which STSGs instead of FTSGs are transplanted on nude mice, results in development of red, raised, and thickened scars that have intrinsic properties closely resembling HTS formation in humans [30]. Although this model is prone to develop scarring, it is highly valuable for investigating several aspects of the wound healing process because of its significant increase in the number of macrophages, mast cells, and fibrocytes [30], along with an increase in biglycan and a decrease in decorin expression in the grafted skin [49]. We found that FLE treatment accelerated re-epithelialization and rete ridges formation in the early stages of healing, which are key parameters in scar pathophysiology [50]. Although scar morphology was not significantly different between FLE-treated and control groups in this dermal fibrotic mouse model, accelerated wound healing is advantageous in itself since is reduces the risk of infections that often complicate the healing process and increases the risk of developing chronic wounds or fibrosis, including HTS. These results complement recent studies investigating the effect of FLE on different phases of wound healing showing accelerated healing of chronic venous leg ulcers, diabetic foot ulcers, and pressure ulcers as well as acute second-degree burns [13,27,28], while highlighting a potential superior effect of FLE in the earlier stages of healing. Further studies are needed to clarify the effect of FLE on reducing the risk of development of scarring. Introducing FLE earlier (before 7 days) after skin grafting might be beneficial and is an interesting aim of a subsequent investigation. In addition, investigating FLE in a model less prone for default development of HTS would also be insightful.

Fibroblastic proliferation and excess collagen deposition are associated with imbalanced healing and scarring [3,51,52]. Interestingly, studies have shown that light within the red part of the visual spectrum suppresses collagen production in human skin fibroblast cultures [51] and inhibits type I collagen expression as well as TGF-β-induced fibroblast to myofibroblast differentiation [53]. Previous studies focusing on FLE have investigated collagen production early after treatment showing that FLE can modulate collagen production both *in vitro* and *in vivo* [12,16]. In the current study, we found that FLE increased collagen 28 days after treatment, whereas it was down-modulated after 56 days. Different types of collagen come into play at different time points during healing; collagen III is produced in the proliferative phase and replaced by collagen I in the remodeling phase [39]. Since the 4-hydroxyproline-assay used does not distinguish between collagen types and thus kinetics of these, more detailed investigations of collagen types are needed to fully understand how FLE controls collagen production in different steps of the wound healing process. However, collagen production alone is inadequate to assess its role in healing, since deposition and remodeling is central for constructing new and fully functional tissues [39,40]. We found that the COI was significantly decreased already at 28 days and remained low at 56 and 84 days after treatment, suggesting that FLE normalizes collagen deposition and remodeling in the tissue in the long-term. Considering that modulation of collagen production is an essential component of any effective tissue repair and anti-fibrotic therapy [54], our data provide support for a new therapeutic modality for managing development of skin fibrosis.

Angiogenesis is vital to restore circulation in the grafted and damaged tissue, and is induced by secreted growth factors, including vascular endothelial growth factor (VEGF) produced by keratinocytes, fibroblasts, and macrophages [37]. While the importance of angiogenesis in the early/proliferative phase of tissue healing is well-documented, less is known about regulation later in the repair process due to the difficulties in monitoring this process *in vivo*. A study has shown that long term overexpression of VEGF-A associates with pathological conditions such as muscle fibrosis [55], underlining the importance of resolving this process later in the wound healing process. We have previously shown that FLE-treatment facilitates angiogenesis, by measuring branching and tube formation in cultures of human aortic endothelial cells 18 h after stimulation *in vitro* [12]. In this study, we found a significant reduction in the number of blood vessels in the grafted tissues 28 and 56 days after FLE stimulation. Earlier, we described that the number of newly formed blood vessels in grafted skin peaked on day 10 and declined again by day 28 after grafting [38]. These observed differences in the effect of FLE on angiogenesis may reflect the different time points when vascularization was assessed after stimulation in each study and that FLE thereby modulates angiogenesis differently in different phases during wound healing.

Although a number of parameters measured in this study were improved by LED light stimulation alone, FLE yielded additional effects demonstrating that the FLE spectrum specifically was critical to the therapeutic benefits observed. Specifically, FLE reduced myofibroblast accumulation whereas Light treatment significantly increased the number of myofibroblasts in the tissue after 56 days. This indicates that blue light directly promotes myofibroblast differentiation which increases the risk of fibrosis whereas the full FLE spectrum reduced myofibroblast numbers. Furthermore, mast cell recruitment and CTGF expression were decreased by FLE in the later phase of repair and remodeling. Combined, these effects resulted in increased wound maturity, which is vital for minimizing the risk of fibrosis in patients [39]. The FLE biophotonic platform utilized in this study has the advantage of generating a broad spectrum of fluorescence within the visible range (440–460 and 500–700 nm) [12,13]. This enables the light to penetrate various layers of the skin and interact with endogenous chromophores, such as flavins/riboflavins, cytochrome c oxidase subunit IV, and nicotinamide adenine dinucleotide hydrogen [56], in different layers in a single treatment. Moreover, previous clinical studies investigating FLE therapy has reported the treatment as applicable, safe, and with few adverse effects [17,18,25,26,27,28], underlining few clinical implications for the use of FLE. For treatment of acute second-degree burns or chronic ulcers, patients did initially experience some pain in relation to the FLE treatment, which was, however, not considered extraordinary compared with the standard of care for these conditions [27,28].

PBM was previously shown to (1) accelerate the healing of formocresol-induced oral ulcers and diabetic wounds in rats due to certain wavelengths stimulating fibroblast proliferation and collagen production [57,58]; (2) induce comparable levels of cell migration and wound closure in a scratch wound model [59]; (3) promote donor site wound healing of the free gingival graft, potentially via reducing reactive oxygen species production [60]; (4) modulate angiogenesis [61]; (5) promote provisional matrix and wound reorganization [60]; and (6) enhance the healing process of third-degree burns in rats [62] and modulate mitochondrial physiology [13] and gene expression [15]. Further experiments are needed to determine which molecular pathways are specifically activated by FLE treatment underpinning its beneficial effects. Potential mechanisms of action likely include photon absorption by endogenous chromophores, photonic energy utilization, and modulation of mitochondrial activity including ATP production, which regulate cellular activation, migration, and protein synthesis that are essential in wound healing [56].

In summary, the aim of this study was to investigate the impact of FLE on tissue repair after skin grafting and examine its therapeutic potential for improving healing and reducing the risk of complication. We found that FLE treatment stimulated healing by increasing re-epithelialization, significantly increasing epidermal thickness and reducing dermal thickness after 28 days, while decreasing number of blood vessels after 56 days. Moreover, collagen production was enhanced at day 28 and significantly reduced at day 56 with COI significantly reduced at all three time points. Finally, mast cells infiltration, myofibroblast formation, and angiogenesis were lowered later in the healing process (84 days after treatment). However, FLE did not directly alter the morphology of the default-developed HTSs in this mouse model.

To conclude, these findings suggest that FLE helps balance the wound healing process at different stages following skin grafting, although a thorough clinical assessment is necessary. This study supports FLE therapy as a possible safe treatment for skin graft patients to help ensure a balanced healing process and a lowered risk of developing chronic wounds.

## References

[1] Sun BK, Siprashvili Z, Khavari PA. Advances in skin grafting and treatment of cutaneous wounds. Science. 2014;346(6212):941–5. 10.1126/science.1253836 25414301

[2] Brown JE, Holloway SL. An evidence-based review of split-thickness skin graft donor site dressings. Int Wound J. 2018;15(6):1000–9. 10.1111/iwj.12967 PMC7949554 30117716

[3] Larouche J, Sheoran S, Maruyama K, Martino MM. Immune regulation of skin wound healing: mechanisms and novel therapeutic targets. Adv Wound Care (N Rochelle). 2018;7(7):209–31. 10.1089/wound.2017.0761 PMC6032665 29984112

[4] Avci P, Gupta A, Sadasivam M, Vecchio D, Pam Z, Pam N, et al. Low-level laser (light) therapy (LLLT) in skin: stimulating, healing, restoring. Semin Cutan Med Surg. 2013;32(1):41–52. PMC4126803 24049929

[5] Anders JJ, Lanzafame RJ, Arany PR. Low-level light/laser therapy versus photobiomodulation therapy. Photomed Laser Surg. 2015;33(4):183–4. 10.1089/pho.2015.9848 PMC4390214 25844681

[6] Hamblin MR. Shining light on the head: photobiomodulation for brain disorders. BBA Clin. 2016;6:113–24. 10.1016/j.bbacli.2016.09.002 PMC5066074 27752476

[7] de Freitas LF, Hamblin MR. Proposed mechanisms of photobiomodulation or low-level light therapy. IEEE J Sel Top Quantum Electron. 2016;22:3. 10.1109/JSTQE.2016.2561201 PMC5215870 28070154

[8] Dancáková L, Vasilenko T, Kováč I, Jakubčová K, Hollý M, Revajová V, et al. Low-level laser therapy with 810 nm wavelength improves skin wound healing in rats with streptozotocin-induced diabetes. Photomed Laser Surg. 2014;32(4):198–204. 10.1089/pho.2013.3586 PMC3985531 24661084

[9] Zhang H, Liu S, Yang X, Chen N, Pang F, Chen Z, et al. LED phototherapy with gelatin sponge promotes wound healing in mice. Photochem Photobiol. 2018;94(1):179–85. 10.1111/php.12816 PMC5771855 28763104

[10] Hamblin MR. Mechanisms and applications of the anti-inflammatory effects of photobiomodulation. AIMS Biophys. 2017;4(3):337–61. 10.3934/biophy.2017.3.337 PMC5523874 28748217

[11] Hamblin MRFC, Huang YY, Freitas de Freitas L, Carroll J. Low-level light therapy: photobiomodulation. Bellingham, WA USA: SPIE PRESS BOOK; 2018, p. 388.

[12] Edge D, Mellergaard M, Dam-Hansen C, Corell DD, Jaworska J, Scapagnini G, et al. Fluorescent light energy: the future for treating inflammatory skin conditions? J Clin Aesthet Dermatol. 2019;12(5):E61–8. PMC6561711 31320979

[13] Scapagnini G, Marchegiani A, Rossi G, Zago M, Jowarska J, Wael M, et al. Management of all three phases of wound healing through the induction of fluorescence biomodulation using fluorescence light energy. Proc. SPIE 10863, Photonic Diagnosis and Treatment of Infections and Inflammatory Diseases II, 108630W (7 March 2019). SPIE; 2019. https://spie.org/Publications/Proceedings/Paper/10.1117/12.2508066.

[14] Zago M, Dehghani M, Jaworska J, Mellergaard M, Edge D, Corell DD, et al. Fluorescent light energy in wound healing: when is a photon something more? Proceedings Volume 11221, Mechanisms of Photobiomodulation Therapy XV; 112210A (2020). San Francisco, California, United States: SPIE BiOS; 2020.

[15] Ferroni L, Zago M, Patergnani S, Campbell SE, Hébert L, Nielsen M, et al. Fluorescent light energy (FLE) acts on mitochondrial physiology improving wound healing. J Clin Med. 2020;9(2):559. 10.3390/jcm9020559 PMC7073965 32085605

[16] Nikolis A, Bernstein S, Kinney B, Scuderi N, Rastogi S, Sampalis JS. A randomized, placebo-controlled, single-blinded, split-faced clinical trial evaluating the efficacy and safety of KLOX-001 gel formulation with KLOX light-emitting diode light on facial rejuvenation. Clin Cosmet Investig Dermatol. 2016;9:115–25. 10.2147/CCID.S100697 PMC4874553 27257391

[17] Antoniou C, Dessinioti C, Sotiriadis D, Kalokasidis K, Kontochristopoulos G, Petridis A, et al. A multicenter, randomized, split-face clinical trial evaluating the efficacy and safety of chromophore gel-assisted blue light phototherapy for the treatment of acne. Int J Dermatol. 2016;55(12):1321–8. 10.1111/ijd.13349 27575854

[18] Nikolis A, Fauverghe S, Scapagnini G, Sotiriadis D, Kontochristopoulos G, Petridis A, et al. An extension of a multicenter, randomized, split-face clinical trial evaluating the efficacy and safety of chromophore gel-assisted blue light phototherapy for the treatment of acne. Int J Dermatol. 2018;57(1):94–103. 10.1111/ijd.13814 29152718

[19] Braun SA, Gerber PA. A photoconverter gel-assisted blue light therapy for the treatment of rosacea. Int J Dermatol. 2017;56(12):1489–90. 10.1111/ijd.13724 28884800

[20] Sannino M, Lodi G, Dethlefsen MW, Nistico SP, Cannarozzo G, Nielsen MCE. Fluorescent light energy: treating rosacea subtypes 1, 2, and 3. Clin Case Rep. 2018;6(12):2385–90. 10.1002/ccr3.1891 PMC6293188 30564333

[21] Koceva I, Rümmelein B, Gerber PA, Edge D, Nielsen MCE. Fluorescent light energy: a new therapeutic approach to effectively treating acne conglobata and hidradenitis suppurativa. Clin Case Rep. 2019;7(9):1769–72. 10.1002/ccr3.2334 PMC6745390 31534746

[22] Mahendran A, Wong XL, Kao S, Sebaratnam DF. Treatment of erlotinib-induced acneiform eruption with chromophore gel-assisted phototherapy. Photodermatol Photoimmunol Photomed. 2019;35(3):190–2. 10.1111/phpp.12446 PMC6850067 30554437

[23] Gerber PA, Scarcella G, Edge D, Nielsen MCE. Biophotonic pretreatment enhances the targeting of senile lentigines with a 694 nm QS-ruby laser. Photodermatol Photoimmunol Photomed. 2020;36(2):159–60. 10.1111/phpp.12518 PMC7079253 31595540

[24] Scarcella GGP, Edge D, Nielsen MCE. Effective removal of solar lentigines by combination of pre- and post- fluorescent light energy treatment with picosecond laser treatment. Clin Case Rep. 2020;8(8):1429–32. 10.1002/ccr3.2839 PMC7455422 32884768

[25] Nikolis AFS, Vezina D, Scapagnini G. Evaluation of biophotonic therapy in a non-healing diabetic foot ulcer: a case report. Diabet Foot Can. 2016;2016(4):25–30.

[26] Nikolis AGD, Pesant Y, Scapagnini G, Vezina D. A prospective case series evaluating the safety and efficacy of the Klox biophotonic system in venous leg ulcers. Chronic Wound Care Manag Res. 2016;3:101–11.

[27] Romanelli M, Piaggesi A, Scapagnini G, Dini V, Janowska A, Iacopi E, et al. Evaluation of fluorescence biomodulation in the real-life management of chronic wounds: the EUREKA trial. J Wound Care. 2018;27(11):744–53. 10.12968/jowc.2018.27.11.744 30398941

[28] Mellergaard M, Fauverghe S, Scarpa C, Pozner VL, Skov S, Hebert L, et al. Evaluation of fluorescent light energy for the treatment of acute second-degree burns. Mil Med. 2021;186(Supplement_1):416–23. 10.1093/milmed/usaa299 33499452

[29] Yang DY, Li SR, Wu JL, Chen YQ, Li G, Bi S, et al. Establishment of a hypertrophic scar model by transplanting full-thickness human skin grafts onto the backs of nude mice. Plast Reconstr Surg. 2007;119(1):104–9. discussion 10-1. 10.1097/01.prs.0000244828.80490.62 17255662

[30] Wang J, Ding J, Jiao H, Honardoust D, Momtazi M, Shankowsky HA, et al. Human hypertrophic scar-like nude mouse model: characterization of the molecular and cellular biology of the scar process. Wound Repair Regen. 2011;19(2):274–85. 10.1111/j.1524-475X.2011.00672.x 21362096

[31] Ding J, Tredget EE. Transplanting human skin grafts onto nude mice to model skin scars. Methods Mol Biol. 2017;1627:65–80. 10.1007/978-1-4939-7113-8_5 28836195

[32] Wang J, Jiao H, Stewart TL, Shankowsky HA, Scott PG, Tredget EE. Increased severity of bleomycin-induced skin fibrosis in mice with leukocyte-specific protein 1 deficiency. J Invest Dermatol. 2008;128(12):2767–76. 10.1038/jid.2008.164 18580965

[33] Tredget EE, Falk N, Scott PG, Hogg AM, Burke JF. Determination of 4-hydroxyproline in collagen by gas chromatography/mass spectrometry. Anal Biochem. 1990;190(2):259–65. 10.1016/0003-2697(90)90190-k 2291469

[34] Junqueira LC, Bignolas G, Brentani RR. Picrosirius staining plus polarization microscopy, a specific method for collagen detection in tissue sections. Histochem J. 1979;11(4):447–55. 10.1007/BF01002772 91593

[35] Osman OS, Selway JL, Harikumar PE, Stocker CJ, Wargent ET, Cawthorne MA, et al. A novel method to assess collagen architecture in skin. BMC Bioinforma. 2013;14(1):260. 10.1186/1471-2105-14-260 PMC3765639 23971965

[36] Martin P, Nunan R. Cellular and molecular mechanisms of repair in acute and chronic wound healing. Br J Dermatol. 2015;173(2):370–8. 10.1111/bjd.13954 PMC4671308 26175283

[37] Guerra A, Belinha J, Jorge RN. Modelling skin wound healing angiogenesis: a review. J Theor Biol. 2018;459:1–17. 10.1016/j.jtbi.2018.09.020 30240579

[38] Shaterian A, Borboa A, Sawada R, Costantini T, Potenza B, Coimbra R, et al. Real-time analysis of the kinetics of angiogenesis and vascular permeability in an animal model of wound healing. Burns. 2009;35(6):811–7. 10.1016/j.burns.2008.12.012 PMC2739287 19423227

[39] Reinke JM, Sorg H. Wound repair and regeneration. Eur Surg Res. 2012;49(1):35–43. 10.1159/000339613 22797712

[40] Bi X, Li G, Doty SB, Camacho NP. A novel method for determination of collagen orientation in cartilage by Fourier transform infrared imaging spectroscopy (FT-IRIS). Osteoarthr Cartil. 2005;13(12):1050–8. 10.1016/j.joca.2005.07.008 16154778

[41] Verhaegen PD, Schouten HJ, Tigchelaar-Gutter W, van Marle J, van Noorden CJ, Middelkoop E, et al. Adaptation of the dermal collagen structure of human skin and scar tissue in response to stretch: an experimental study. Wound Repair Regen. 2012;20(5):658–66. 10.1111/j.1524-475X.2012.00827.x 22882499

[42] van Zuijlen PP, de Vries HJ, Lamme EN, Coppens JE, van Marle J, Kreis RW, et al. Morphometry of dermal collagen orientation by Fourier analysis is superior to multi-observer assessment. J Pathol. 2002;198(3):284–91. 10.1002/path.1219 12375260

[43] Hinz B. Formation and function of the myofibroblast during tissue repair. J Invest Dermatol. 2007;127(3):526–37. 10.1038/sj.jid.5700613 17299435

[44] Wilgus TA, Wulff BC. The importance of mast cells in dermal scarring. Adv Wound Care (N Rochelle). 2014;3(4):356–65. 10.1089/wound.2013.0457 PMC3985512 24757590

[45] Lian N, Li T. Growth factor pathways in hypertrophic scars: Molecular pathogenesis and therapeutic implications. Biomed Pharmacother. 2016;84:42–50. 10.1016/j.biopha.2016.09.010 27636511

[46] Morris DE, Wu L, Zhao LL, Bolton L, Roth SI, Ladin DA, et al. Acute and chronic animal models for excessive dermal scarring: quantitative studies. Plast Reconstr Surg. 1997;100(3):674–81. 10.1097/00006534-199709000-00021 9283567

[47] Dunkin CSJ, Pleat JM, Gillespie PH, Tyler MPH, Roberts AHN, McGrouther DA. Scarring occurs at a critical depth of skin injury: Precise measurement in a graduated dermal scratch in human volunteers. Plastic Reconstr Surg. 2007;119(6):1722–32. 10.1097/01.prs.0000258829.07399.f0 17440346

[48] Zhu KQ, Engrav LH, Gibran NS, Cole JK, Matsumura H, Piepkorn M, et al. The female, red Duroc pig as an animal model of hypertrophic scarring and the potential role of the cones of skin. Burns. 2003;29(7):649–64. 10.1016/s0305-4179(03)00205-5 14556722

[49] Momtazi M, Kwan P, Ding J, Anderson CC, Honardoust D, Goekjian S, et al. A nude mouse model of hypertrophic scar shows morphologic and histologic characteristics of human hypertrophic scar. Wound Repair Regen. 2013;21(1):77–87. 10.1111/j.1524-475X.2012.00856.x 23126488

[50] Arno AI, Gauglitz GG, Barret JP, Jeschke MG. Up-to-date approach to manage keloids and hypertrophic scars: a useful guide. Burns. 2014;40(7):1255–66. 10.1016/j.burns.2014.02.011 PMC4186912 24767715

[51] Mamalis A, Siegel D, Jagdeo J. Visible red light emitting diode photobiomodulation for skin fibrosis: key molecular pathways. Curr Dermatology Rep. 2016;5:121–8. 10.1007/s13671-016-0141-x PMC4848333 27182462

[52] Biernacka A, Dobaczewski M, Frangogiannis NG. TGF-beta signaling in fibrosis. Growth Factors. 2011;29(5):196–202. 10.3109/08977194.2011.595714 PMC4408550 21740331

[53] Sassoli C, Chellini F, Squecco R, Tani A, Idrizaj E, Nosi D, et al. Low intensity 635 nm diode laser irradiation inhibits fibroblast-myofibroblast transition reducing TRPC1 channel expression/activity: new perspectives for tissue fibrosis treatment. Lasers Surg Med. 2016;48(3):318–32. 10.1002/lsm.22441 26660509

[54] McDougall S, Dallon J, Sherratt J, Maini P. Fibroblast migration and collagen deposition during dermal wound healing: mathematical modelling and clinical implications. Philos Trans A Math Phys Eng Sci. 2006;364(1843):1385–405. 10.1098/rsta.2006.1773 16766351

[55] Karvinen H, Pasanen E, Rissanen TT, Korpisalo P, Vähäkangas E, Jazwa A, et al. Long-term VEGF-A expression promotes aberrant angiogenesis and fibrosis in skeletal muscle. Gene Ther. 2011;18(12):1166–72. 10.1038/gt.2011.66 21562595

[56] Lubart R, Lavi R, Friedmann H, Rochkind S. Photochemistry and photobiology of light absorption by living cells. Photomed Laser Surg. 2006;24(2):179–85. 10.1089/pho.2006.24.179 16706696

[57] Lau PS, Bidin N, Krishnan G, Nassir Z, Bahktiar H. Biophotonic effect of diode laser irradiance on tensile strength of diabetic rats. J Cosmetic Laser Ther. 2015;17(2):86–9. 10.3109/14764172.2014.968587 25260140

[58] de Carvalho FB, Andrade AS, Rasquin LC, de Castro IV, Cangussu MC, Pinheiro AL, et al. Effect of laser (lambda 660 nm) and LED (lambda 630 nm) photobiomodulation on formocresol-induced oral ulcers: a clinical and histological study on rodents. Lasers Med Sci. 2015;30(1):389–96. 10.1007/s10103-014-1680-7 25354753

[59] Spitler R, Berns MW. Comparison of laser and diode sources for acceleration of in vitro wound healing by low-level light therapy. J Biomed Opt. 2014;19(3):38001. 10.1117/1.JBO.19.3.038001 24638250

[60] Wang CY, Tsai SC, Yu MC, Lin YF, Chen CC, Chang PC. Light-emitting diode irradiation promotes donor site wound healing of the free gingival graft. J Periodontol. 2015;86(5):674–81. 10.1902/jop.2015.140580 25630628

[61] de Sousa AP, Paraguassú GM, Silveira NT, de Souza J, Cangussú MC, dos Santos JN, et al. Laser and LED phototherapies on angiogenesis. Lasers Med Sci. 2013;28(3):981–7. 10.1007/s10103-012-1187-z 22923269

[62] de Vasconcelos Catão MH, Nonaka CF, de Albuquerque RL Jr, Bento PM, de Oliveira, Costa R. Effects of red laser, infrared, photodynamic therapy, and green LED on the healing process of third-degree burns: clinical and histological study in rats. Lasers Med Sci. 2015;30(1):421–8. 10.1007/s10103-014-1687-0 25391372

